# On the Use of the Jackknife to Compare R_XY_ Statistics in Conservation Genomics Research

**DOI:** 10.1111/1755-0998.70148

**Published:** 2026-05-11

**Authors:** Oliver Patrick Stuart

**Affiliations:** ^1^ School of Biological, Earth, and Environmental Sciences University College Cork Cork Ireland

**Keywords:** biological statistics, bootstrap, confidence intervals, conservation genetics, conservation genomics, jackknife

## Abstract

Many useful quantities in conservation genomics are point estimates that summarise genetic variation at the population or species level. This presents a challenge to the application of null hypothesis testing frameworks, which require some measure of uncertainty to test the authenticity of measured group differences. The jackknife is a convenient statistical resampling tool which sees regular use to this end: producing confidence intervals for point estimates. Unfortunately, the slightly obscure nature of the jackknife coupled with its superficial resemblance to the bootstrap has led to its misuse, specifically in the analysis of R_XY_ statistics, a recently formulated quantity designed to measure relative mutation load. In this review, I summarise the application of the jackknife as a method to produce confidence intervals and review its uses in the conservation/population genomics literature. I outline how it is similar to and different from the bootstrap, and how the treatment of jackknife pseudo‐values as bootstrap pseudo‐values produces inflated confidence in otherwise negligible group differences. I then provide suggestions for the future use of the jackknife in conservation genomics.

## 1 Introduction

In conservation and population genomics we are usually interested in summarising some aspect of genetic variation at the population or species level. To this end, there are summary statistics at our disposal which can be used to measure population differentiation and inbreeding (Malécot 1948; Wright 1943, 1949), genetic load (Do et al. 2015; Grossen et al. 2020), introgression (Durand et al. 2011; Green et al. 2010; Patterson et al. 2012; Rosenzweig et al. 2016), and myriad other biological processes and quantities. Calculating these statistics requires summing or multiplying values across many individuals and/or loci, for example in statistics that take population allele frequencies as inputs. As a result, large datasets comprising complex genetic polymorphism data from multiple individuals and populations are often distilled into point estimates. Abstraction and simplification are obviously necessary for comparable inferences to be made across systems. However, such estimates present a challenge to working scientists who overwhelmingly favour null hypothesis testing frameworks to evaluate the authenticity of group differences. These tests require some measure of uncertainty, for example a sample standard error which allows the use of Student's *t* distribution for a test of differences in means. To obtain uncertainty measures, statistical resampling of individuals or loci is often employed.

The jackknife, a resampling procedure with superficial similarities to but that predates the bootstrap, has risen in popularity in the last decade as a convenient method for calculating the variance in genome‐wide summary statistics. Jackknife resampling is routinely used in the analysis of introgression (Patterson et al. 2012), effective population size (Do et al. 2014; Waples and Do 2008), and genetic load (Do et al. 2015). In this latter case, Do et al. (2015) used a block jackknife procedure to estimate confidence intervals for R_XY_, a complex ratio statistic that measures relative genetic load among populations. They noted that they used the jackknife replicates to calculate standard errors “using jackknife theory”, although they did not describe exactly what formulae were used. Since then, numerous studies have used jackknifing improperly, treating the distribution of jackknife pseudo‐values as if it represents the empirical error or variance of R_XY_ (e.g., Chen, Feng, et al. 2025; Dussex, Kurland, et al. 2023; Furni et al. 2025; Mathur et al. 2022; von Seth et al. 2022). In one case, it appears as if jackknife pseudo‐values were used themselves as samples for statistical testing (Grossen et al. 2020) resulting in inflated significance of group differences. It is high time that we have some updated reference which guides the correct use of this statistical procedure, in the context of R_XY_ and otherwise.

In this article, I briefly outline the use of the jackknife as a statistical procedure specifically in the context of conservation genetics/genomics. I use simulations to demonstrate its properties and to compare it to the bootstrap. I then describe how it has been misused in the literature, mostly in the analysis of R_XY_ statistics. I do not aim to overturn the results of any of the studies cited herein, but simply to draw attention to the improper interpretation of the jackknife and to strengthen the analytical rigour with which conservation genomics research is conducted. I then demonstrate the correct use of the jackknife to estimate standard errors using a published example of R_XY_ statistics (Stuart et al. 2025) and compare it to a bootstrap approach.

## 2 The Jackknife in Practice

The jackknife is a non‐random leave‐one‐out procedure originally proposed by Quenouille (1956) and further developed by Tukey (1958) which can be used to estimate the bias or variance of a statistical estimator (which I hereafter refer to simply as a statistic). Here, I focus primarily on its use in estimating the sample variance of a statistic i.e., the construction of confidence intervals. To perform the jackknife on a dataset of *n* observations, one simply removes each observation in turn and recalculates the statistic with the *n*−1 observations, resulting in *n* pseudo‐values. The mean of these pseudo‐values is an accurate estimator of the true sample mean (Miller 1974) but modified formulae are required for the variance and other higher order moments. Tukey proposed in a conference abstract (Tukey 1958) that these pseudo‐values could be treated as independent and identically distributed random variables. The pseudo‐values would therefore have an approximate *t* distribution with *n*−1 degrees of freedom (or an approximate Z distribution for large enough *n*). The jackknife could then be used for the estimation of confidence intervals for otherwise intractable statistics:

$${\mathrm{SE}}_{\text{jacknife}}=\sqrt{\frac{n-1}{n}\sum_{j=1}^{n}{\left(x_{-j}-\bar{x}\right)}^{2}}$$

where *n* is the sample size, $x_{-j}$ is the jackknife pseudo‐value of the statistic omitting the *j* ^th^ observation, and $\bar{x}$ is the average of estimators across the jackknife pseudo‐values. This formula resembles the standard deviation (which is used to calculate the standard error of a statistic from by bootstrapping; see below) except for the sample size correction of $\frac{n-1}{n}$. The exact notation used here is based on an essay by McIntosh (2016). While Tukey (1958) is credited with inspiring the use of the jackknife for interval estimation, he himself did not publish any specific equations to this purpose.

The use of the jackknife in population genetics dates originally to Mueller (1979) who compared it to the delta method (which involves taking a Taylor series expansion of an expectation) for estimating the variance of Nei's genetic distance (Nei 1972). He found that, in general, the jackknife performed favourably to the delta method in terms of reducing the bias associated with sampling small numbers of loci. The method was then shortly taken up to estimate the variance in co‐ancestry coefficients (Reynolds et al. 1983) and *F*‐statistics (Weir and Cockerham 1984). Crucial to the resampling process is the assumption of linkage equilibrium among loci (Weir and Cockerham 1984). In the context of genome‐wide polymorphism data, the physical linkage of loci (under which condition total linkage equilibrium is unlikely) motivates block resampling. Loci in close physical proximity on a chromosome share similar evolutionary histories as they tend to be inherited together as long haplotypes. Resampling of large segments of chromosome (and the loci contained therein) aims to reduce some of the resultant correlation among loci by treating a block of linked loci as a single observation. In this case, the *n* observations are grouped into *g* blocks of contiguous loci. The jackknife proceeds similarly in this case, where the statistic is recalculated without the loci contained in block *g*, resulting in *g* pseudo‐values. Each pseudo‐value is then weighted by the proportion of *n* used to calculate it. This modified procedure, known as the weighted block jackknife, was originally proposed in a more general context by Busing et al. (1999). They provided modified equations for mean and sample standard error of an estimator using the weighted block jackknife:

$${\hat{\theta }}_{\text{weighted block jackknife}}=g\hat{\theta }-\sum_{j=1}^{g}\frac{\left(n-m_{j}\right){\hat{\theta }}_{-j}}{n}$$

$${\mathrm{SE}}_{\text{weighted block jackknife}}=\sqrt{\frac{1}{g}\sum_{j=1}^{g}\left(\frac{n-m_{j}}{m_{j}}\right){\left(\hat{\theta }-{\hat{\theta }}_{-j}\right)}^{2}}$$

where *g* is the number of blocks, *n* is the total number of observations (i.e., the total number of loci summed across all blocks), $\hat{\theta }$ is the value of the statistic calculated using the entire dataset, ${\hat{\theta }}_{-j}$ is the value of the statistic omitting the *j* ^th^ block, and *m_j_* is the number of observations (loci, in our case) in the the *j* ^th^ block.

The best documented examples of the weighted block jackknife in the population genetics literature come from the study of archaic introgression in human and Neandertal populations. Reich et al. (2009) used the jackknife standard error to calculate a Z‐score for *f*‐statistics (not to be confused with *F*‐statistics) estimated from single nucleotide polymorphism data comparing contemporary Indian populations. The authors warned that the assumption of normality in Z‐scores from jackknife standard errors is often violated when $\left|Z\right|>2$, meaning that large Z‐scores can be treated as statistically significant but that standard Z tables cannot be used to convert these into *p* values (Reich et al. 2009; Thorburn 1977). The authors did not show any results to this effect. The weighted block jackknife was then used in the analysis of the first draft Neandertal genome (Green et al. 2010) to obtain standard errors for *D‐*statistics measuring the extent of introgression between Neandertals and contemporary human populations. They varied the number of blocks and found that a minimum block size of 2 Mb was large enough to obtain stable jackknife standard errors. Reich et al. (2009) instead used 5 cM blocks rather than measuring by physical distance. Green et al. (2010) likely chose to use physical distance to define blocks because the extent of linkage disequilibrium (LD) in the Neandertal genome was not known at the time.

To my knowledge, there is no literature concerning how best to define blocks for resampling genomic datasets, and Green et al. (2010) are the only authors to present an investigation of the effect of varying genomic block sizes for jackknifing. The optimal size will probably vary depending on the physical extent of LD in the genomes of the populations being studied. Patterson et al. (2012) used the weighted block jackknife to compute standard errors for several quantities, *D* and *f*‐statistics among them (now incorporated into the software ADMIXTOOLS), and used a variety of block sizes: 5 cM, 10 cM, even whole chromosomes. The use of whole chromosomes as blocks was taken up by later authors (e.g., Grossen et al. 2020) although it is unclear how this approach differs from using even block sizes. It is possible to mathematically correct for the unevenness of blocks (Busing et al. 1999) but extreme length variation and small chromosome numbers in some species may not yield robust standard errors using the weighted block jackknife. A physically explicit (in the chromosomal sense) simulation study would be illuminating to this point: how much does chromosome structure alter the robustness of a whole‐chromosome jackknife procedure?

Another area where the jackknife sees regular use is the study of genetic load. There has been a boom in the study of deleterious mutations in the conservation genetics literature over the last decade, largely driven by the declining costs of sequencing technologies (Dussex, Morales, et al. 2023; Robinson et al. 2023; van Oosterhout 2020), and it is now *de rigueur* to calculate some measure of the genetic load for the populations being studied. A statistic which has seen rising use to this effect is R_XY_ (Do et al. 2015) which was formulated explicitly to compare deleterious mutation load among human populations. The calculation of R_XY_ follows from counts of alleles in two populations (Do et al. 2015). A reference population Y is chosen to which a focal population X is compared. The expected probability of sampling an allele in X but not Y given a random sample of one haploid genome from each population (averaging over all possible pairs of samples) is calculated and summed across *i* sites:

$$L_{X,\;\text{not}\;Y}=\sum_{i}\left(\frac{d_{X}^{i}}{n_{X}^{i}}\right)\left(1-\frac{d_{Y}^{i}}{n_{Y}^{i}}\right)$$

where *n* is the number of sampled haploid genomes and *d* is the count of alleles from that sample. Alleles can be polarised to the ancestral state or unpolarised, but of course the interpretation will differ depending on whether *d* refers to a count of derived versus non‐reference alleles. R_XY_ is then calculated as a ratio of the L_X, not Y_ statistic to its inverse, where the focal and reference populations are flipped:

$$R_{\mathrm{XY}}=L_{X,\;\text{not}\;Y}/L_{Y,\;\text{not}\;X}$$

If the probability of sampling an allele (derived or non‐reference) at a given site is the same in both X and Y, then R_XY_ = 1. Values less than or greater than 1 are taken as evidence for a depletion or enrichment in population X compared to population Y, and therefore as evidence of increased or reduced efficacy of selection acting on the sites included. Meaningful comparisons are made by comparing values among populations or mutation classes.

Do et al. (2015) used a weighted block jackknife, dividing the human genome into 100 contiguous blocks and estimating standard errors for R_XY_ from the jackknife pseudo‐values. In the conservation genetics literature, the use of R_XY_ also traces back to Xue et al. (2015) who referred to it as R_A/B_ and also used a jackknife procedure to estimate the variance of the statistic. Crucially, neither of these papers contained equations for their standard error calculations, nor did they cite literature where such equations might be found. For the other cases discussed above, *f‐*statistics and *D*‐statistics, the jackknife procedure is built into existing software tools, sidestepping individual user error. This is not the case for R_XY_, a statistic which remains relatively underexamined. As a result, numerous subsequent studies have used the jackknife to produce confidence intervals for R_XY_ which are implausibly narrow, likely because they use incorrect equations (in some cases, available computer code indicates that this is indeed the case). I describe the specifics of this error below in section “The jackknife is not the bootstrap”.

The final major area where the jackknife is used for confidence interval estimation is in the analysis of effective population size (N_e_). Here, the resampling approaches differ slightly to the previous examples. Single sample approaches to contemporary N_e_ estimation with molecular data typically make use of LD (reviewed by Waples 2024). Non‐independence among measurements is inherent to the calculation of LD using more than two loci: among *L* loci there are $\frac{L\left(L-1\right)}{2}$ unique pairs but only $\frac{L}{2}$ non‐overlapping pairs. In the LDNE software, Waples and Do (2008) implemented a jackknife which operates over pairs of loci rather than individual loci, and used this to estimate the effective sample size of pairs based on equations from Hill (1981). This effective sample size is the degrees of freedom required to calculate confidence intervals for the average LD among locus pairs.

The estimation of confidence intervals for N_e_ is one of the few areas of population/conservation genomics where the use of the jackknife as a statistical procedure has been recently evaluated. Jones et al. (2016) suggest that jackknifing over locus pairs is not jackknifing at all as no recalculation is performed, rather the existing LD calculations are re‐averaged. They used simulations to show that jackknifing over individuals produces confidence intervals that more effectively capture true N_e_ under realistic data conditions such as when working with massive single‐nucleotide polymorphism datasets. This discrepancy, between the jackknife over individuals and what Jones et al. (2016) refer to as the “pseudo‐jackknife” over loci, may also apply to other published examples of the jackknife, including the weighted block jackknife. This label, the “pseudo‐jackknife” (although I will continue to refer to resampling of loci/blocks as a jackknife), does not necessarily imply that the method cannot be used to produce viable confidence intervals. The conclusions of Reynolds et al. (1983), that the jackknife does indeed capture the true variance in co‐ancestry coefficients, were based on a comparison to the true variances obtained from simulated data, although they did not opt to resample individuals as a comparison to resampling of loci. To my knowledge, the only examples aside from Jones et al. (2016) that resample individuals rather than loci do so in the context of single locus analyses, e.g., van Dongen and Backeljau's (1995) bootstrap tests of inbreeding coefficients. In this case, resampling of individuals is the only option.

This problem is not new and was discussed (although in slightly different terms) by Weir (1996) and briefly by Weir and Cockerham (1984). The question at stake here is: exactly which variance is being explored when resampling is performed? Weir (1996) referred to two sources of variance, genetic variance and statistical variance. Genetic variance refers to variance across loci attributable to stochasticity in the evolutionary process (Weir and Cockerham (1984) focused on randomness in gamete formation). Statistical variance refers to the sampling variance that affects our ability to adequately characterise a given realisation of the evolutionary process. It would be informative to compare the effects of jackknifing over individuals rather than loci or blocks of loci in the estimation of quantities like R_XY_ or *D*‐statistics, indeed any statistic that is calculated using many loci simultaneously. This would be intractable for some workflows, for example when using *angsd* (Korneliussen et al. 2014) which would require the generation of large intermediate analysis files for each resample. However, genomic analyses that operate on hard‐called genotypes may be amenable to this approach.

## 3 Wherefore the Jackknife?

The primary alternative to the jackknife is the bootstrap, another resampling procedure which was originally proposed by Efron (1979). In the original proposition, Efron (1979) showed that the jackknife is in fact a linear approximation of the bootstrap, and so the two are closely related. Bootstrapping proceeds by randomly resampling observations from a dataset with replacement to produce a new dataset of equivalent size to the original, recalculating the statistic of interest with each resampled dataset to produce a distribution of pseudo‐values. In this way it superficially resembles the jackknife, although the properties of the two differ.

Efron (1979) suggested that the bootstrap is more generalisable and reliable than the jackknife. Indeed, other authors have cautioned that although the jackknife is convenient, its performance as an estimator for confidence intervals is not assured (Miller 1974). A convenient property of the bootstrap is that the standard deviation of the distribution of pseudo‐values is an estimator of the sample standard error (Efron 1979), allowing the easy calculation of confidence intervals for summary statistics. The standard deviation is a familiar formula for anyone who has taken even an elementary statistics course. Why, then, should anyone prefer the more mathematically unwieldy jackknife?

The simple answer is computational tractability. In the case of block resampling of genomic datasets, each block may consist of thousands to tens of thousands of loci and the generation of each bootstrap resample may be time consuming. Compared to the jackknife, where each resampled dataset is produced by simply removing one of the observations, the bootstrap may take some time. For effective population size estimates from LD statistics, this may be even more intractable given that the number of unique locus pairs grows much faster than the number of loci.

Setting aside the size of the resampled datasets, the total number of bootstraps to perform need not be that large. If the criterion is a stable estimate of the standard error of a statistic, then this could be easily verified on an *ad hoc* basis by tracking the coefficient of variation as the number of bootstraps increases. Indeed, the introduction of such stopping criteria has been proposed in the analysis of phylogenetic trees (Pattengale et al. 2010), wherein 100–500 bootstraps were deemed adequate for stable node support estimates in maximum likelihood phylogenetic inference. The number of bootstraps required to produce stable results (regardless of the statistic being calculated) is rarely studied in population/conservation genetic research.

Finally, the issue of “pseudo‐jackknifing” over loci and not samples will also apply to bootstrap resampling. However, if a bootstrap of genomic windows or loci is considered more costly than a jackknife, then a bootstrap of individuals may exceed even that in computation time.

## 4 The Jackknife is Not the Bootstrap

In my opinion, the superficial similarity of the bootstrap to the jackknife, in that they both involve recalculation of a statistic on subsets of data to produce a distribution of pseudo‐values, has led to the misuse of the jackknife in the conservation genomics literature. Specifically, this has led to false confidence in the precision of R_XY_ estimates. It is my concern that this false confidence may lead to unfounded conclusions regarding the mutation load in threatened and vulnerable populations, and that this practice may spread to the use of other statistics.

**FIGURE 1:**
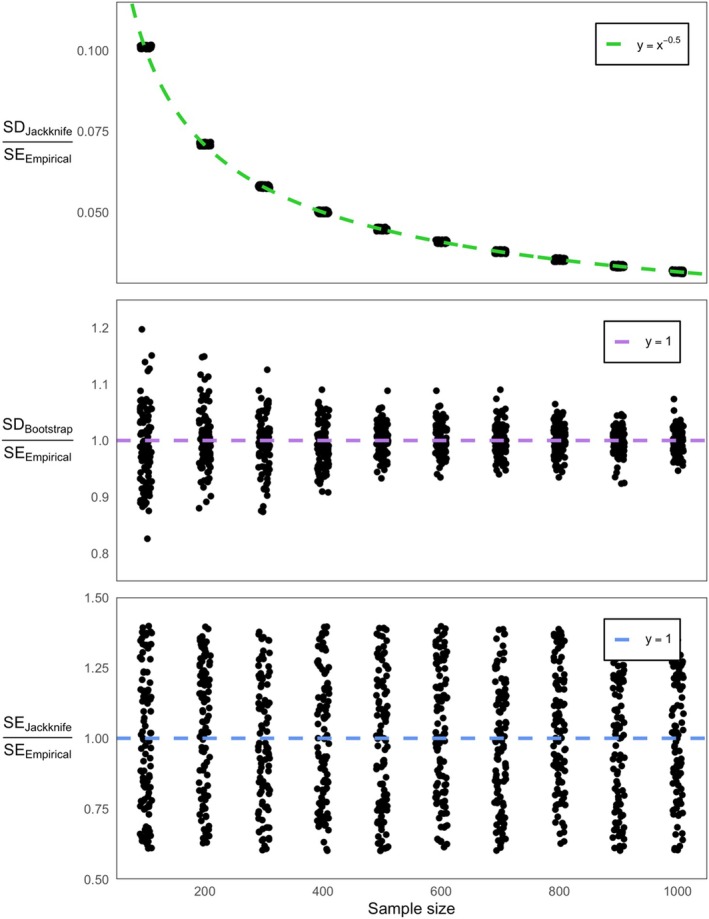
Comparison of the jackknife and the bootstrap for estimating the precision of a summary statistic, in this case the mean. For all panels, samples were simulated from a normal distribution with mean and standard deviation equal to 10 and 1. For each random sample of size *n* (x‐axis), I calculated estimators of the standard error based on the standard deviation of the jackknife (top), the standard deviation of the bootstrap (middle), and the standard error of the jackknife (bottom) following the calculation in McIntosh (2016) and then divided each by the empirical standard error to estimate bias.

From a verbal description alone, we can tell that the distribution of pseudo‐values obtained using the jackknife will be much tighter than for the bootstrap. If we were to take a sample of 100 measurements from a population and perform a jackknife to estimate our confidence in the mean, then each jackknife resample will contain 99% of the original data and produce a pseudo‐value closely resembling the true sample mean. Simple simulations can be used to demonstrate that the standard deviation of jackknife pseudo‐values will systematically underestimate the sample standard error (of the mean, in this example). This bias increases with the number of observations following a simple power law relationship of $y=x^{-0.5}$, where y is the relative underestimation and x is the number of samples used for the jackknife (Figure 1, top). With 100 observations, the standard deviation of jackknife pseudo‐values will be one tenth of the true standard error (Figure 1, top). The bootstrap is not biased in this way. The standard deviation of bootstrap pseudo‐values is an accurate estimator of the sample standard error with precision commensurate to the number of resamples taken (Figure 1, middle). While the standard deviation of jackknife pseudo‐values cannot be directly substituted for the sample standard error in the same way as for the bootstrap, the formula above produces estimates of the standard error that tend towards the true value with a precision that is insensitive to the sample size (Figure 1, bottom).

Many of the papers citing Do et al. (2015) for the use of the R_XY_ statistic also use the jackknife to estimate the statistical significance of such differences. Notably, Grossen et al. (2020) used this procedure to analyse R_XY_ values for mutations of different predicted impacts on protein function in bottlenecked populations of domestic alpine ibex ( *Capra ibex* ). To produce pseudo‐values, they used whole chromosomes as blocks (*n* = 29 for the domestic goat assembly used; Dong et al. 2013) and performed tests of Tukey's Honest Significant Difference (HSD) comparing the jackknife pseudo‐values of R_XY_ among different mutation classes. They concluded that bottlenecked populations, relative to stable ones, purged highly deleterious mutations but accumulated weakly deleterious ones given that R_XY_ values calculated from pseudo‐values were significantly lower or higher respectively than a non‐coding mutation class. The use of jackknife pseudo‐values as if they represented independent samples from the population violates the Tukey's HSD test assumptions and fails to recognise the distinct properties of the jackknife as a statistical procedure. This is also, as far as I am aware, the first instance of jackknife pseudo‐values themselves being presented graphically (fig. 2e in Grossen et al. 2020). Since this study was published, numerous other studies have performed jackknife procedures for R_XY_ and presented boxplots of the pseudo‐values or their mean ± 1–2 standard deviations as if they represented the sample variance of R_XY_ (e.g., Chen, Zhang, et al. 2025; Dussex, Kurland, et al. 2023; Furni et al. 2025; Lan et al. 2025). The result is a set of exceedingly narrow distributions/confidence intervals which are then taken as evidence for the enrichment/depletion of deleterious mutations in threatened populations. It is not my aim here to dispute the findings of the above cited studies which all presented multiple lines of evidence in support of their conclusions. However, as I have shown above with reference to the literature and my own simulations, the jackknife requires particular treatment to produce accurate estimates of uncertainty.

## 5 A Worked Example

Here, I reanalyse an empirical dataset (Stuart et al. 2025) to demonstrate how (1) the plotting of jackknife pseudo‐values and (2) the incorrect use of jackknife pseudo‐values in a null hypothesis test can both lead to false confidence in group differences. The data are drawn from captive and wild populations of *Dryococelus australis*, the Lord Howe Island stick insect. Allele frequencies for mutations in both populations were estimated with low‐coverage whole genome sequence data, and fitness effects of mutations were approximated by estimating their predicted impact on protein function with gene models from Stuart et al. (2023) using snpEff (Cingolani et al. 2012); a similar procedure to Grossen et al. (2020). Mutations are grouped into high, moderate, and low impact categories, with an additional category for non‐coding mutations in intergenic regions. Impacts on protein function are assumed to have unconditionally negative fitness consequences. The question I am interested in is: “has the strong bottleneck at the onset of the captive breeding program resulted in the purging or enrichment of deleterious mutations?” I answered this by calculating R_XY_ for the four different mutation categories in the captive individuals, using the wild individuals as the reference population. I standardised these values using the intergenic mutations, such that if a mutation class is experiencing the same average effect of selection as intergenic mutations, then R_XY_ = 1. The whole‐genome values strongly suggest that highly impactful mutations were purged (R_XY_ = 0.73), likely because of increased homozygosity in captivity exposing them to selection, and there may also be some accumulation of mutations in the low (R_XY_ = 1.01) and moderate (R_XY_ = 1.01) categories. This is almost identical to the patterns seen by Grossen et al. (2020).

To estimate intervals around these values, I divided the genome into 100 contiguous blocks of roughly equal numbers of loci (uneven chromosome/scaffold length means that exactly equal blocks cannot be defined). I then resampled these blocks using a jackknife procedure and calculated R_XY_ pseudo‐values. I did the same for the 16 autosomes of the *D. australis* genome, using each chromosomal scaffold as a block. I used the Busing et al. (1999) equations to calculate means and standard errors. Finally, I used the 100 contiguous blocks to calculate a bootstrap estimate of the standard error, using standard equations for the weighted mean and standard deviation. I generated resampled datasets using 100, 500, and 1000 bootstraps to assess the stability of standard errors.

**FIGURE 2:**
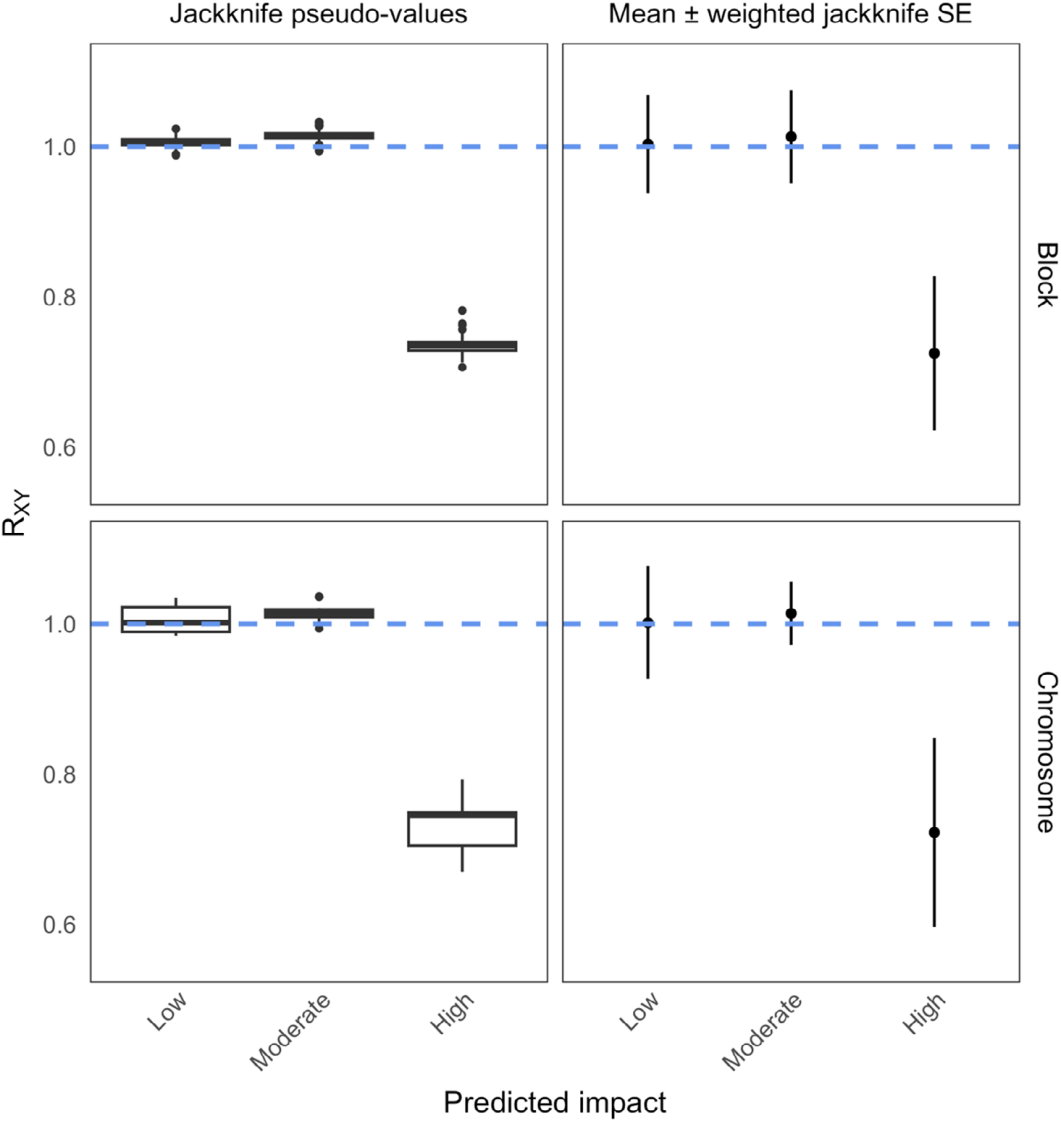
Comparison of methods for estimating and visualising variance in R_XY_. Left column: Boxplots showing the distribution of R_XY_ pseudo‐values from each jackknife replicate. Right column: Mean R_XY_ ± 1 standard error using the equations formulated for a weighted block jackknife. Top row: Jackknifing performed over 100 roughly equally sized contiguous blocks across the *D. australis* autosomal genome. Bottom row: Jackknifing performed using the 16 *D. australis* autosomes as blocks. The hashed blue line indicates R_XY_ = 1, the neutral expectation.

**FIGURE 3:**
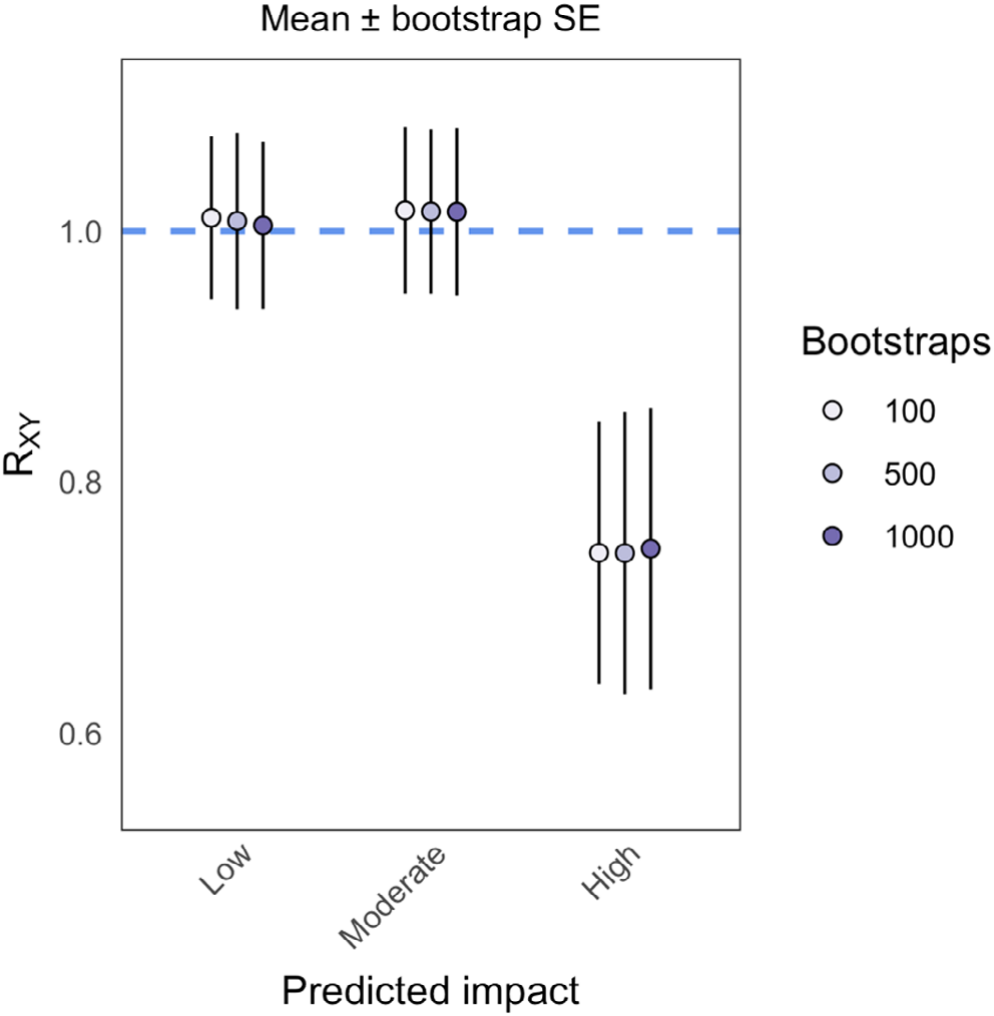
Results of block bootstrapping for estimating variance in R_XY_. Points represent weighted mean R_XY_ for each mutation impact category, error bars represent one standard error (calculated as the weighted standard deviation of R_XY_ across bootstrap pseudo‐values), and colours denote the number of bootstrap resamples taken. The hashed blue line indicates R_XY_ = 1, the neutral expectation.

The distributions of R_XY_ from the equal size block jackknife are exceedingly narrow (Figure 2, top‐left), but slightly less narrow for whole chromosomes (Figure 2, bottom‐left). For equal length blocks, the distributions of R_XY_ for both low and moderate impact mutations only slightly overlap 0. For the whole chromosome jackknife, this is also true for the moderate impact mutations but not the low impact mutations. Mean R_XY_ for low and moderate impact mutations was clearly less than one standard error away from 1. This was true for both the equal length block and chromosome jackknife (Figure 2, top‐right, bottom‐right). In all cases, neither the distributions nor the mean ± 1 standard error overlap 1 for the high impact mutations. This is strong evidence that the captive *D. australis* population has purged highly impactful mutations. The bootstrap estimates of the standard error reflect those of the block jackknife with the Busing et al. (1999) equations (Figure 3). Both mean R_XY_ and the size of the confidence intervals varied slightly with the number of bootstrap replicates, although the magnitude of these fluctuations was minor (Figure 3).

The calculation of standard errors (for the bootstrap and the jackknife) suggests that only high impact mutations have been affected by the captivity bottleneck, but based on the raw distributions of R_XY_ from jackknife replicates (Figure 2, left), it seems plausible that the captive population has simultaneously accumulated moderate and/or low impact mutations. How do these apparent visual differences stand up to a hypothesis test?

I performed two variations on a *t*‐test to test the alternative hypothesis that R_XY_ for each mutation class was different from the neutral expectation. In some datasets, the chromosome number may be small, and so I deemed a *t*‐test a more realistic test than a Z‐test for these cases where the degrees of freedom would be low. In all cases, I used a two‐sided test. First, I used the jackknife pseudo‐values of R_XY_ as samples in a paired *t*‐test. I compared each protein‐coding mutation class to intergenic mutations. I can use a paired *t*‐test because each respective pseudo‐value for the two classes being compared was calculated using the same jackknife replicate. Next, I used the means and standard errors for both the jackknife and the bootstrap to test whether the standardised R_XY_ for each category was different to 1 in a single sample *t*‐test.

The *t*‐tests of equal sized block jackknife pseudo‐values were uniformly significant, suggesting a depletion of highly impactful mutations and an enrichment of low and moderate impact mutations in captivity relative to intergenic mutations (Table 1). High and moderate impact mutations were significantly different from intergenic mutations when using chromosome jackknife pseudo‐values (Table 1). In all cases, *t* values were much greater than when using the means and standard errors, regardless of whether the test was significant. This underscores the fact that using jackknife pseudo‐values as samples will overestimate the significance of group differences, as suggested by the visual comparison (Figure 2). Only the high impact mutation class was significantly different from 1 when using the means and standard errors to perform the *t*‐test (Table 2). Block and chromosome jackknife and bootstrap test statistics were more similar when using means and standard errors (Table 2) than when using the pseudo‐values (Table 1), suggesting some of the bias produced by uneven block size and different block numbers can be corrected for. The number of bootstrap replicates used did not impact the test results, although there was a slight trend towards 0 in the *t* values for all impact classes with more bootstraps (Table 2).

**TABLE 1:** Results of paired two‐sided *t*‐tests testing the alternative hypothesis that *R*_XY_ (captive individuals relative to wild individuals) for three protein‐coding mutation types is different to intergenic mutations, indicating a relative enrichment (*t* > 0) or depletion (*t* < 0). Samples of pseudo‐values were generated by a block jackknife, where the blocks were either 100 contiguous segments of chromosome containing roughly equal numbers of loci throughout the *D. australis* autosomes or the 16 autosomal scaffolds themselves. Bolded values indicate significant tests at *α* = 0.05.

Variant effect	Mean difference	SE difference	*t*	*p*
Block (*n* = 100)				
Low	0.005	0.001	8.760	**0.000**
Moderate	0.012	0.001	21.411	**0.000**
High	−0.209	0.001	−235.422	**0.000**
Chromosome (*n* = 16)				
Low	0.005	0.004	1.355	0.196
Moderate	0.012	0.002	5.007	**0.000**
High	−0.209	0.007	−29.12	**0.000**

**TABLE 2:** Results of two‐sided single‐sample *t*‐tests testing the alternative hypothesis that standardised R_XY_ (captive individuals relative to wild individuals, standardised using intergenic mutations) for a given protein‐coding mutation class is significantly different from 1, indicating a relative enrichment (*t* > 0) or depletion (*t* < 0). Means and standard errors were calculated using a block jackknife/bootstrap procedure. The blocks were either 100 contiguous segments of chromosome (“Block”) containing roughly equal numbers of loci throughout the *D. australis* autosomes or the 16 autosomal scaffolds themselves (“Chromosome”). For the jackknife, equations from Busing et al. (1999) were used to calculate means and standard errors. For the bootstrap, the weighted mean and weighted variance were used.

Variant effect	Mean	SE	*t*	*p*
Block Jackknife (*n* = 100)				
Low	1.003	0.065	0.056	0.956
Moderate	1.013	0.062	0.215	0.831
High	0.725	0.103	−2.679	**0.009**
Chromosome Jackknife (*n* = 16)				
Low	1.002	0.075	0.026	0.980
Moderate	1.014	0.042	0.336	0.742
High	0.722	0.126	−2.200	**0.044**
Block Bootstrap (*n* = 100)				
Low	1.011	0.065	0.167	0.868
Moderate	1.017	0.066	0.249	0.804
High	0.744	0.105	−2.443	**0.016**
Block Bootstrap (*n* = 500)				
Low	1.008	0.070	0.116	0.908
Moderate	1.016	0.065	0.239	0.811
High	0.743	0.112	−2.284	**0.023**
Block Bootstrap (*n* = 1000)				
Low	1.005	0.067	0.069	0.945
Moderate	1.016	0.067	0.232	0.816
High	0.747	0.112	−2.250	**0.025**

It is worth mentioning that the small sample size of chromosomes in the *D. australis* genome (16 autosomes) makes for both an underpowered *t*‐test and a less egregious underestimation of the sample standard error: a greater number of observations produces downward bias in the variance of jackknife pseudo‐values (Figure 1). Therefore, the more chromosomes an organism has, the more downwardly biased a chromosome block jackknife procedure will be unless the correct equations are used. However, it is likely that this bias also occurs in the other direction: the fewer chromosomes an organism has, the more likely there is to be upward bias in the size of confidence intervals.

## 6 Conclusions and Recommendations

I have demonstrated (rather, redemonstrated), using simple simulations and empirical data, that the jackknife can be used to estimate the sampling variance of genomic statistics and therefore can be used to test for group differences. Specific equations (Busing et al. 1999) for the standard error and the mean are crucial for this. My hope is that readers will be more attuned to the subtleties of the jackknife as a variance estimation procedure, one which is convenient and robust (if sometimes conservative) when the pseudo‐values are treated correctly. Alternatively, the bootstrap, an equally convenient and more robust procedure, stands ready for use without the need for specialised equations (except for the weighting for uneven block size). Historical issues regarding the computational tractability of the bootstrap become less concerning as the speed of computation grows. Researchers should not shy away from the bootstrap just because it may take more time than expected.

I put forward the following recommendations regarding the use of resampling procedures to test for group differences in genomic statistics:

1. Divide the genome into contiguous blocks with roughly equal numbers of loci, as in Do et al. (2015) when resampling loci in genomic datasets. Using chromosomes as blocks is convenient but inter/intraspecific variation in chromosome number and size may affect the relative performance of the block jackknife.
2. Perform a bootstrap over the blocks and use the weighted mean and weighted variance to estimate standard errors for statistical testing.
3. If using the block jackknife instead, use the equations presented above (Busing et al. 1999) to calculate means and standard errors.
4. Proceed normally through data visualisation and statistical testing using the means and standard errors. Do not use the jackknife pseudo‐values; these will almost always artificially inflate precision and overestimate group differences.

## Funding

This work was supported by Grant‐Aid Agreement no. PDOC/23/04/01, administered by the Marine Institute *Foras na mara* and funded under the Marine Research Programme by the Government of Ireland.

## Conflicts of Interest

The author declares no conflicts of interest.

## Data Availability

No new data were generated in the production of this manuscript. All code and allele frequency data required to conduct the analyses and reproduce the figures herein are available at https://github.com/OliverPStuart/2025_Jackknife_Review/. The full code required to process the original raw sequence data, calculate allele frequencies, and estimate the effects of mutations on protein function is available at https://github.com/OliverPStuart/Daustralis_Purging_Manuscript/.
